# A circular RNAs dataset landscape reveals potential signatures for the detection and prognosis of early-stage lung adenocarcinoma

**DOI:** 10.1186/s12885-021-08293-7

**Published:** 2021-07-06

**Authors:** Zhiying Chen, Jiahui Wei, Min Li, Yongjuan Zhao

**Affiliations:** Respiratory Department, The Third Hospital of Jilin University, No. 126. Xiantai Street, Changchun, 130033 Jilin China

**Keywords:** Lung adenocarcinoma, Circular RNAs, Survival analysis, Functional analysis, Gene–drug interaction analysis

## Abstract

**Background:**

This study aimed to identify potential circular ribonucleic acid (circRNA) signatures involved in the pathogenesis of early-stage lung adenocarcinoma (LAC).

**Methods:**

The circRNA sequencing dataset of early-stage LAC was downloaded from the Gene Expression Omnibus database. First, the differentially expressed circRNAs (DEcircRNAs) between tumour and non-tumour tissues were screened. Then, the corresponding miRNAs and their target genes were predicted. In addition, prognosis-related genes were identified using survival analysis and further used to build a network of competitive endogenous RNAs (ceRNAs; DEcircRNA–miRNA–mRNA). Finally, the functional analysis and drug–gene interaction analysis of mRNAs in the ceRNA network was performed.

**Results:**

A total of 35 DEcircRNAs (30 up-regulated and 5 down-regulated circRNAs) were identified. Moreover, 135 DEcircRNA–miRNA and 674 miRNA–mRNA pairs were predicted. The survival analysis of these target mRNAs revealed that 60 genes were significantly associated with survival outcomes in early-stage LAC. Of these, high levels of *PSMA 5* and low levels of *NAMPT, CPT 2* and *TNFSF11* exhibited favourable prognoses. In addition, the DEcircRNA–miRNA–mRNA network was constructed, containing 5 miRNA–circRNA (hsa_circ_0092283/hsa-miR-762/hsa-miR-4685-5p; hsa_circ_0070610/hsa-let-7a-2-3p/hsa-miR-3622a-3p; hsa_circ_0062682/hsa-miR-4268) and 60 miRNA–mRNA pairs. Functional analysis of the genes in the ceRNA network showed that they were primarily enriched in the Wnt signalling pathway. Moreover, *PSMA 5, NAMPT, CPT 2* and *TNFSF11* had strong correlations with different drugs.

**Conclusion:**

Three circRNAs (hsa_circ_0062682, hsa_circ_0092283 and hsa_circ_0070610) might be potential novel targets for the diagnosis of early-stage LAC.

## Background

Lung cancer remains a serious public health problem across the world with a relatively high mortality rate (approximately 1.7 million deaths each year) [1]. Currently, nearly 80% patients undergoing primary lung cancer have been diagnosed with non-small cell lung cancer (NSCLC). Lung adenocarcinoma (LAC) is a common subtype of non-small cell lung cancer and accounts for around 40% of all lung cancer cases [2]. Although surgical resection has been the most efficient therapy option for early-stage LAC, the reported 5-year survival rates for patients remains unsatisfactory (< 15%) [3]. Therefore, there is an urgent need to identify effective diagnostic signatures correlated with the initiation and development of early-stage LAC.

Circular RNAs (circRNAs) are a subclass of non-coding covalent closed circular RNAs generated by alternative splicing [4]. Overwhelming evidence has shown that circRNAs are evolutionarily conserved and function as miRNA sponges or competitive endogenous RNAs (ceRNAs) to regulate gene transcription [5]. In recent decades, an increasing number of studies have suggested that circRNAs may play crucial roles in cancers, such as hepatocellular carcinoma and NSCLC [6, 7]. Furthermore, microarray-based RNA microarray profiling contributes substantially to systematically screen promising biomarkers that are involved in cancer progression [8]. Zhu et al analysed a circRNA microarray dataset and found that 59 circRNAs had differential expression in LAC samples than in non-tumour samples. Further, they noted that in most cases, up-regulated hsa_circ_0013958 was strongly related to the TNM stage and lymphatic metastasis, indicating that this circRNA might be a potential target for the early detection of LAC [9]. Chen et al profiled three non-coding RNAs expression datasets of NSCLC and emphasised that hsa_circ_0078767/hsa_miR-330-3p/ *RASSF1A* axis served significant roles in cell proliferation and invasion of NSCLC [10]. A previous study performed a circRNA microarray analysis of early-stage LAC using GSE101684 set and identified 357 differentially expressed circRNAs (DEcircRNAs). Furthermore, the altered expression of circRNA (hsa_circRNA_404833) was validated using real-time quantitative reverse transcription polymerase chain reaction (qRT-PCR) methods and is predicted to interact with miR-149-5p that was associated with LAC development [11]. However, a comprehensive bioinformatics analysis based on this dataset has not been conducted.

Herein, we re-analysed the circRNA microarray dataset (GSE101684) to identify novel diagnostic and prognostic biomarkers for the management of early-stage LAC. The DEcircRNAs were extracted between tumour and non-tumour tissues; thereafter, predictive analyses of miRNAs and their target genes were performed. The survival analysis was performed to identify prognosis-related genes; then, the DEcircRNA–miRNA–mRNA network was constructed. Finally, the functional analysis and drug–gene interaction analysis were performed to screen novel therapeutic targets for LAC treatment. We believe that our findings will provide new insights into the involvement of circRNAs in the pathogenesis of early-stage LAC.

## Methods

### Data source and DEcircRNA screening

The circRNA expression data (GSE101684) of early-stage LAC and the corresponding annotation files were downloaded from the National Centre for Biotechnology Information Gene Expression Omnibus (NCBI-GEO) repository (http://www.ncbi.nlm.nih.gov/geo/). This dataset contained eight samples (four tumour tissues and paired adjacent normal tissues of patients with early-stage LAC) and was generated using the GPL21825 074301 Arraystar Human CircRNA microarray V2 sequencing platform. Then, the raw circRNA expression data were pre-processed using the R limma package, including background correction, normalisation and concentration prediction [12]. The probes were annotated to the corresponding circRNAs by combing the matrix data with the platform annotation files. If multiple probes mapped to the same circRNA, the average value of these probes was considered as the expression value of the circRNA. Linear model-experience Bayesian statistics using the limma package in R combined with t-tests were used for nonspecific filtration of the expression profile data, and the DEcircRNAs were determined. The cut-off criteria of the adjusted *P*-value (adj. *P*-value) was set at 0.05, and the criterion of fold change was set at |log_2_ FC | > 1.5. The heatmap and volcano plot of the DEcircRNAs were drawn using R pheatmap and ggplot2 package, respectively.

### Prediction of miRNAs regulated by DEcircRNAs and miRNA–target genes

First, the circRNAs and human mature miRNAs sequencing files with FASTA format were obtained on the basis of the circBase and miRBase databases. Then, miRanda (version 3.3a; https://omictools.com/miranda-tool) was used to predict the interactions between miRNAs and circRNAs. The miRNA*–*DEcircRNA pairs were further extracted with a score value of > 150 and an energy value of <− 30 and visualised using Cytoscape. Subsequently, we obtained the top five circRNAs in the miRNA*–*DEcircRNA regulatory network as per the degree. Furthermore, the top 5_circRNA–miRNA network was constructed, and the miRNAs in this sub-network were screened using a threshold score value of ≥170. Following this, miRWalk 2.0 (http://zmf.umm.uni-heidelberg.de/apps/zmf/mirwalk2/) was employed to predict the target genes of extracted miRNAs above based on seven databases (miRWalk, miRanda, miRanda, miRDB, miRMap, Pictar2, RNA22 and Targetscan) [13]. In order to further screen the LAC-related miRNA–mRNA pairs in the obtained miRNA–mRNA pairs, we took the intersection of the targeted genes (mRNA) in the previous step and the LAC-related genes included in the CTD database [14]. Finally, the miRNA/LAC-associated mRNA interactions were determined.

### Survival analyses

The mRNA data and relevant clinical information of early-stage LAC samples were first downloaded from The Cancer Genome Atlas (TCGA) database, and these data were labelled as the TCGA dataset. Then, the expression value and survival information of mRNAs in miRNA/LAC-related mRNA network were extracted from the TCGA dataset. All the mRNAs were split into the high-expression and low-expression groups as per median expression, using the R survival package (version 2.42–6; https://cran.r-project.org/web/packages/survival/index.html) [15]. Moreover, the overall survival (OS) was computed using the Kaplan–Meier (KM) survival method to assess the prognostic value of the LAC-related genes. The correlation coefficient *p* < 0.05 showed that mRNA was significantly related to prognosis.

### Construction of DEcircRNA–miRNA–mRNA network and functional analysis

In the final step, we obtained the mRNAs that were significantly associated with prognosis. We screened the miRNA–mRNA pairs related to these mRNAs. Then, we integrated these relationship pairs with the previously obtained circRNA–miRNA relationship pairs. Using the Cytoscape, we constructed a lncRNA–miRNA–mRNA network. Further, the Kyoto Encyclopedia of Genes and Genomes (KEGG) enrichment analysis and Gene Ontology–Biological Process category (GO-BP) functional annotation analysis of these genes were performed using the R clusterprofiler package [16–18]. We have only showed the top five GO-BPs and pathways.

### Drug–gene interaction analysis

The Drug–Gene Interaction database (DGIdb) is an online resource and provides gene druggability information [19]. We used the DGIdb (version 2.0; http://www.dgidb.org/) database to predict the relationships between drugs and genes in the ceRNA network [20]. The FDA and DrugBank were selected as drug databases and the interaction type of NA was excluded from this study. Finally, the drug–gene network was built using Cytoscape.

#### circRNA detection using qRT-PCR

Total RNA from all the LAC tissues (20) and paired adjacent tissues (20) was extracted using TRIzol Reagent (Invitrogen, Carlsbad, CA, USA). Quantity and quality of RNA were determined spectrophotometrically at 260 and 280 nm, respectively. The integrity and contamination were confirmed using denaturing agarose gel electrophoresis. qRT-PCR methods were performed using an ABI ViiA7 utilising SYBR Premix Ex Taq II (Tli RNaseH Plus) (Takara) as per the manufacturer’s instructions. Primers (Table 1) were synthesised by Sangon Biotech (Shanghai, China). The data were analysed using the comparative cycle threshold (ΔCT) method after three independent experiments. All the results are expressed as the mean ± standard deviation (SD) values.

**Table 1:** Premiers used in QRT-PCR

Primer	sequences(5′-3′)
hsa_circ_0062682-hF	TGCCTCACCAAGTGGAACAA
hsa_circ_0062682-hR	GGGCTTCAGCGACAGGTT
hsa_circ_0092283-hF	ACGGCAGAGCTGGCCTTGGA
hsa_circ_0092283-hR	AGGAAGGTGGCAGCAGGA
hsa_circ_0070610-hF	GCTGGACAAAGGATGACG
hsa_circ_0070610-hR	GATGGGCTTGGTAGGTGA
GAPDH-hF	TGACAACTTTGGTATCGTGGAAGG
GAPDH-hR	AGGCAGGGATGATGTTCTGGAGAG

## Results

### Identification of DEcircRNAs

**Fig. 1:**
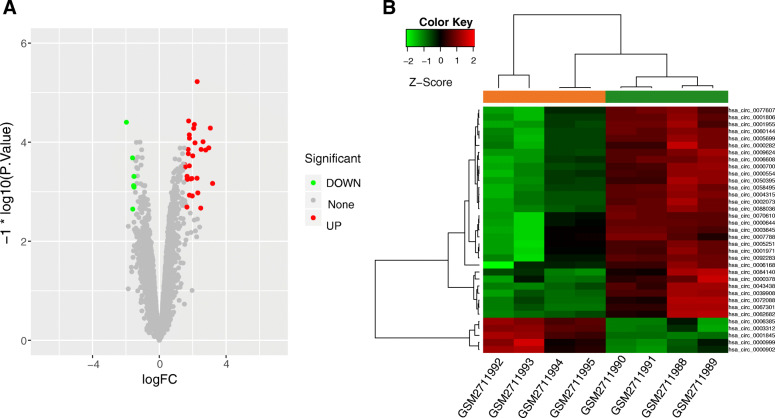
The differential expression analysis of circRNAs. **a** The volcano plot of differentially expressed circRNAs. Red and green dots respectively represent the up-regulated circRNAs and down-regulated circRNAs. **b** The heatmap of differentially expressed circRNAs

A total of 35 DEcircRNAs (30 up-regulated DEcircRNAs and 5 down-regulated DEcircRNAs) were identified from the LAC tumour tissues and the adjacent normal tissues as per the screening criteria described in the Methods section. As shown in Fig. 1, the clustering analysis revealed that these DEcircRNAs significantly discriminated LAC and healthy control samples.

### Predicting DEcircRNA–miRNA and miRNA–target gene pairs

**Fig. 2:**
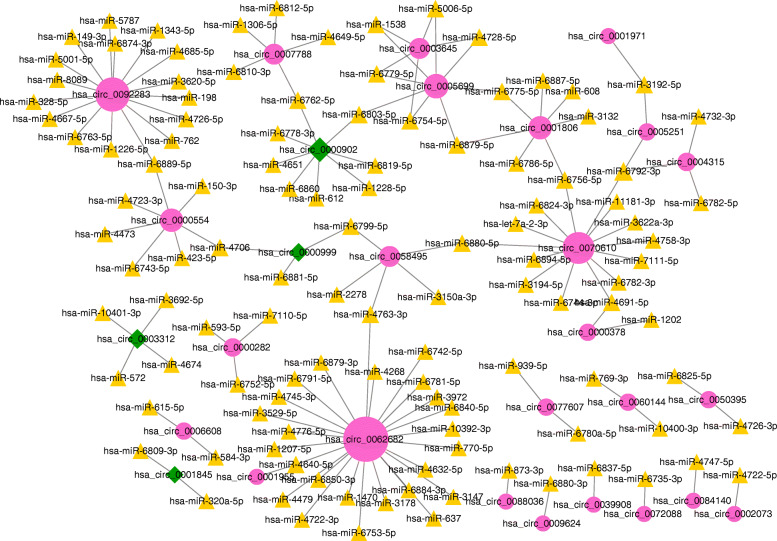
The differentially expressed circRNA–miRNA regulatory network. Red and green nodes separately represent up-regulated circRNAs and down-regulated circRNAs. The yellow triangles show the predicted miRNAs. A larger node represents a higher degree

The miRNAs targeted by DEcircRNAs were first predicted as per the above-mentioned methods. The results indicated that there were 135 DEcircRNA–miRNA pairs among 25 up-regulated DEcircRNAs, 4 down-regulated DEcircRNAs, and 115 miRNAs (Fig. 2). Then, the top five DEcircRNAs in the DEcircRNA–miRNA network were extracted, including up-regulated hsa_circ_0062682, up-regulated hsa_circ_0092283, up-regulated hsa_circ_0070610, up-regulated hsa_circ_0005699 and down-regulated hsa_circ_0000902. Then, 772 miRNA–target gene pairs were determined using the prediction of miRWalk2.0. Meanwhile, the LAC-related genes were also downloaded from the CTD database. Finally, a total of 674 miRNA–gene interactions were obtained by screening the overlapped genes between target genes in the miRNA–target gene network and the LAC-related genes.

### Survival analysis

**Fig. 3:**
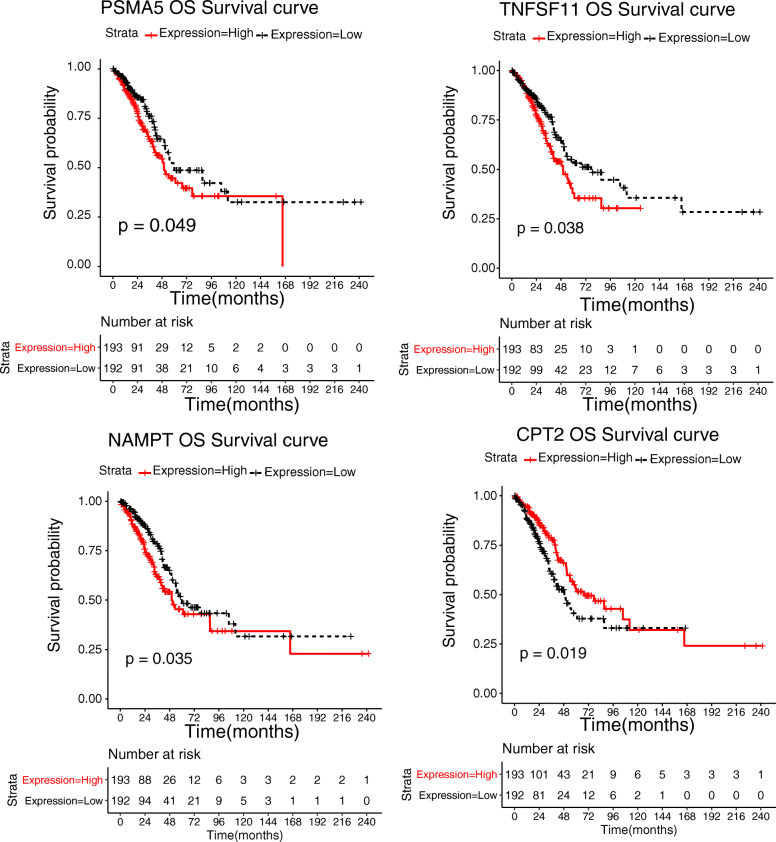
The Kaplan–Meier survival curves of the following four genes: *PSMA 5*, *TNFSF11*, *NAMPT* and *CPT2*. The horizontal axis represents the overall survival time. The vertical axis indicates the survival ratio. The red and black lines represent the high-expression group and low-expression group, respectively

To further evaluate the diagnostic values of LAC-related genes in the miRNA–gene network, we downloaded the expression data and clinical information of early-stage LAC patients from the TCGA database. We only included the data of early-stage LAC in the TCGA dataset. A total of 385 samples were used for the final analyses. Thus, the survival analysis of these LAC-related genes was performed; the results showed that 60 genes were closely correlated with the clinical outcomes of LAC patients. The KM survival curves of four genes are shown in Fig. 3, including Proteasome α5 subunit (*PSM 5*), tumour necrosis factor superfamily member 11 (*TNFSF11*), Nicotinamide phosphoribosyltransferase (*NAMPT*) and carnitine palmitoyltransferase 2 (*CPT2*). We noted that the up-regulated *TNFSF11, PSM 5* and *NAMPT* were strongly associated with worse prognosis. However, higher expression levels of *CPT 2* exhibited favourable survival outcomes.

### CircRNA–miRNA–mRNA network and functional analyses

**Fig. 4:**
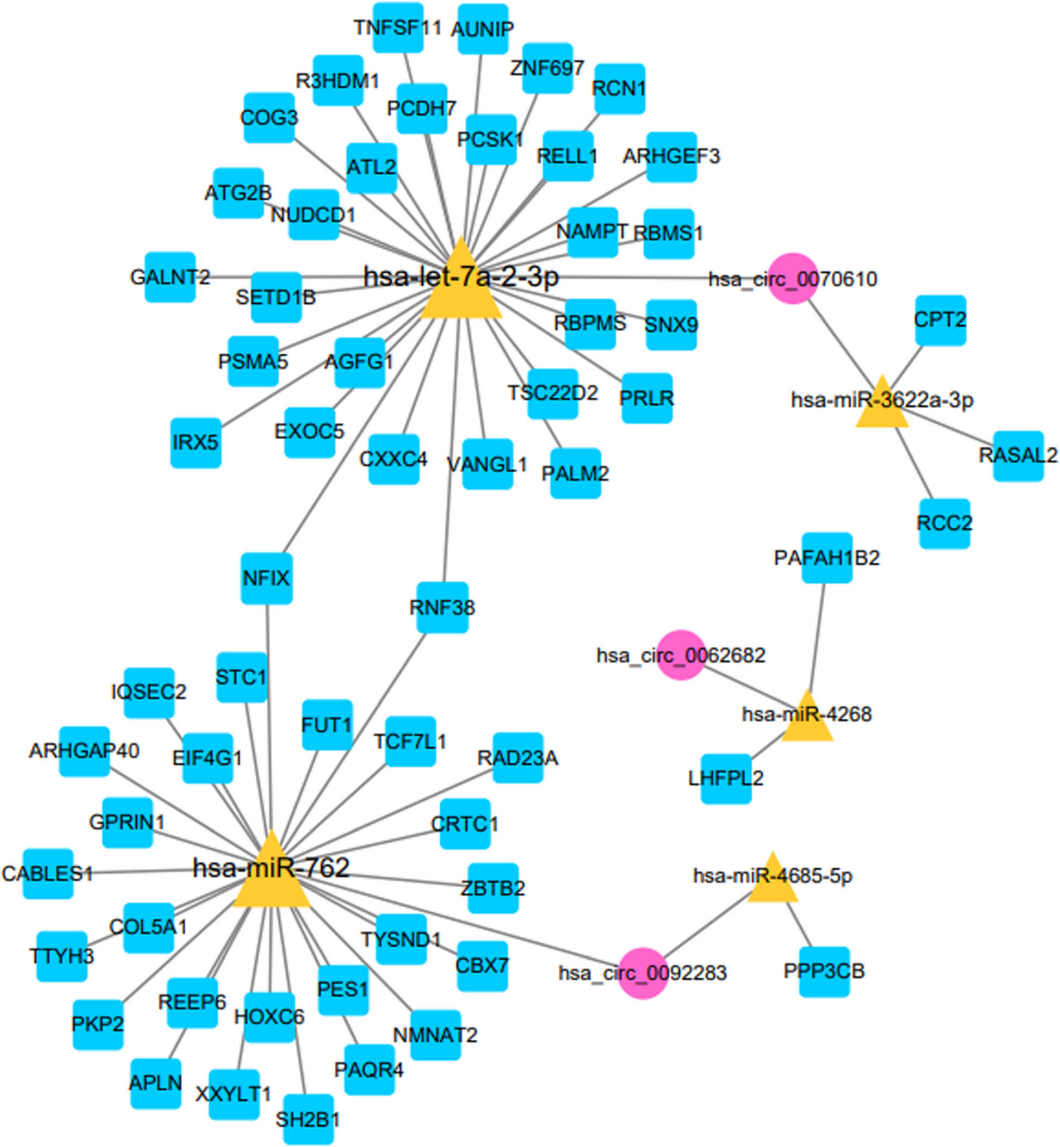
The differentially expressed circRNA–miRNA–mRNA regulatory network. The red nodes represent up-regulated circRNAs, the yellow triangles represent the miRNAs and the blue squares indicate prognosis-related mRNAs. A larger node represents a higher degree

**Fig. 5:**
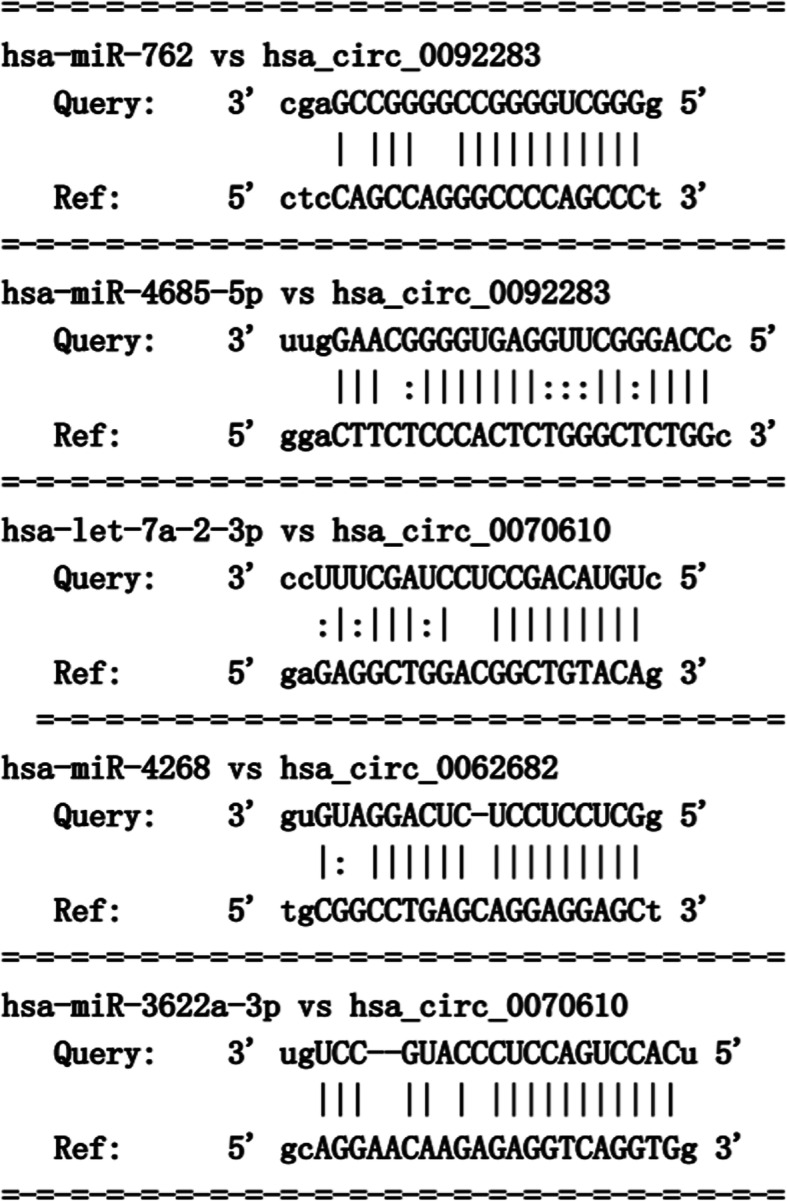
The map of the binding sites of miRNAs and circRNAs

**Fig. 6:**
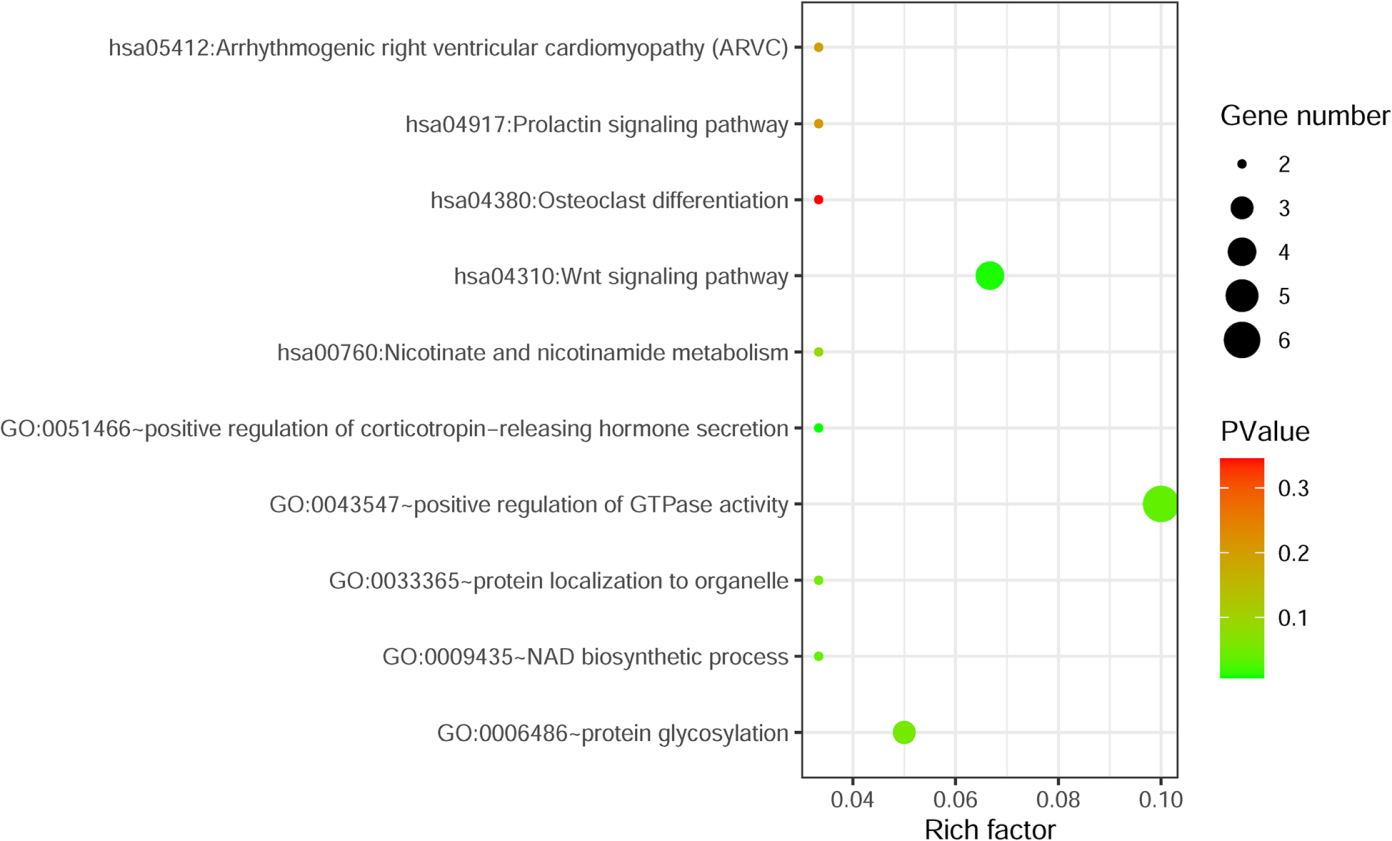
The functional analysis of differentially expressed circRNAs. The dot size shows the generation, and the colour, ranging from red to green, indicates the increasing significance

A total of 62 miRNA–mRNA pairs and 5 miRNA–circRNA pairs were used to build the circRNA–miRNA–mRNA network that contained 5 miRNAs (hsa-miR-762, hsa-miR-4685-5p, hsa-let-7a-2-3p, hsa-miR-4268 and hsa-miR-3622a-3p), 3 up-regulated circRNAs (hsa_circ_0062682, hsa_circ_0092283 and hsa_circ_0070610) and 60 prognosis-related genes (Fig. 4). The prediction analysis of miRNA-binding sites suggested that hsa_circ_0092283 interacted with two miRNAs (hsa-miR-762 and hsa-miR-4685-5p), whereas hsa_circ_0070610 bound strongly to hsa-let-7a-2-3p and hsa-miR-3622a-3p. Meanwhile, hsa_circ_0062682 exhibited a close relationship with has-miR-4268 (Fig. 5). Specific GO categories, such as positive regulation of GTPase activity and protein glycosylation, were significantly enriched in these DE genes (DEGs) (Fig. 6). Moreover, the results of KEGG analysis revealed the potential biological relationship between our gene set and the Wnt signalling pathway (Fig. 6).

### Drug–gene interaction prediction

**Fig. 7:**
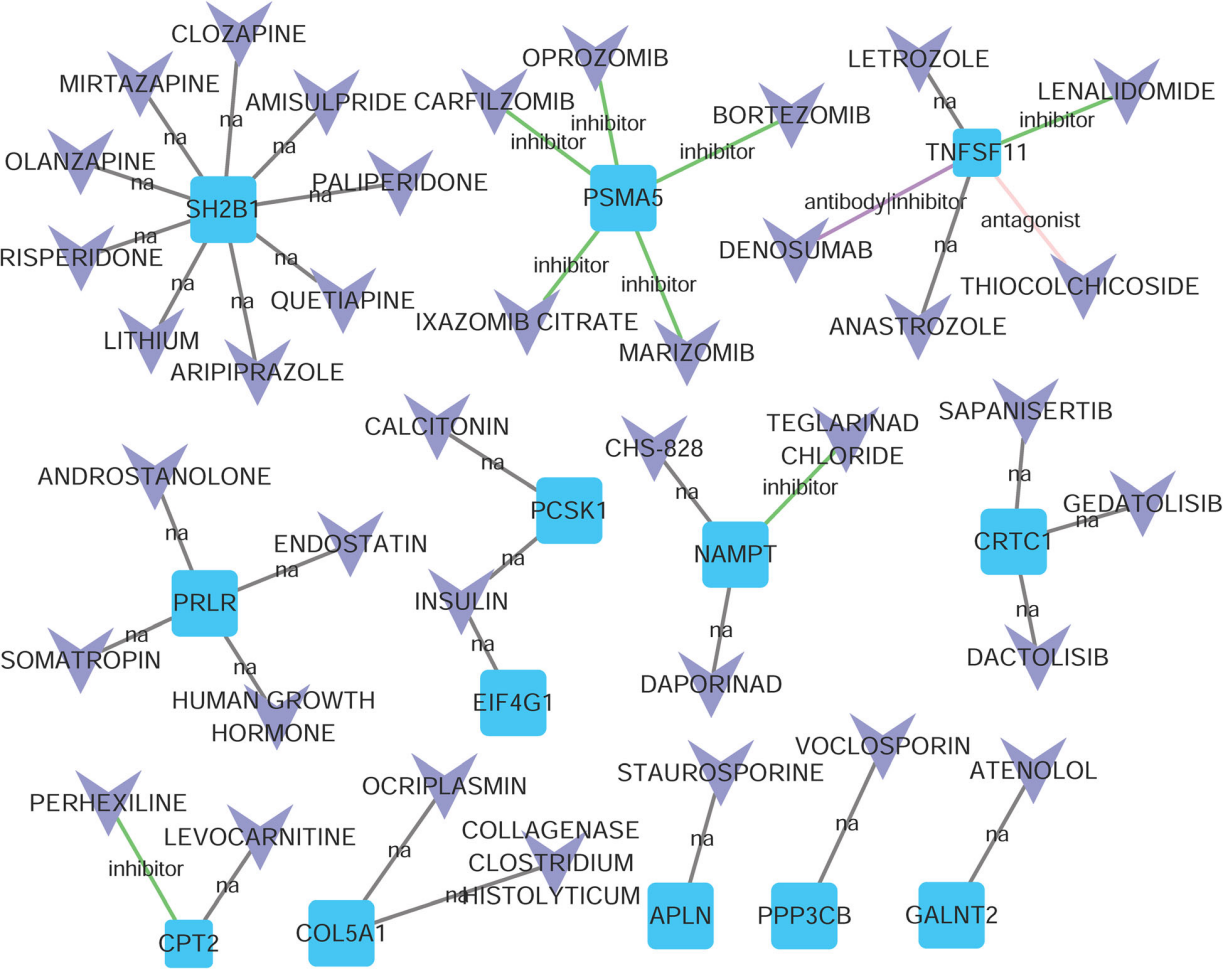
The drug–gene interaction network. The squares depict the genes, and the V nodes represent the drug molecules

Our predictive analysis of drug–gene interactions showed that there were 39 drug–gene pairs among 13 genes and 26 drug molecules. As shown in Fig. 7, we can explore the relationship between some genes and drugs.

Furthermore, *PSM 5* showed close correlation with the following five drugs: carfilzomib, bortezomib, oprozomib, ixazomib citrate and marizomib. They are all inhibitors. *TNFSF11* was associated with lenalidomide, thiocolchicoside and denosumab. Teglarinad chloride was an inhibitor for NAMPT. Moreover, *CPT2* closely interacted with perhexiline.

### Verification of key circRNAs

**Fig. 8:**
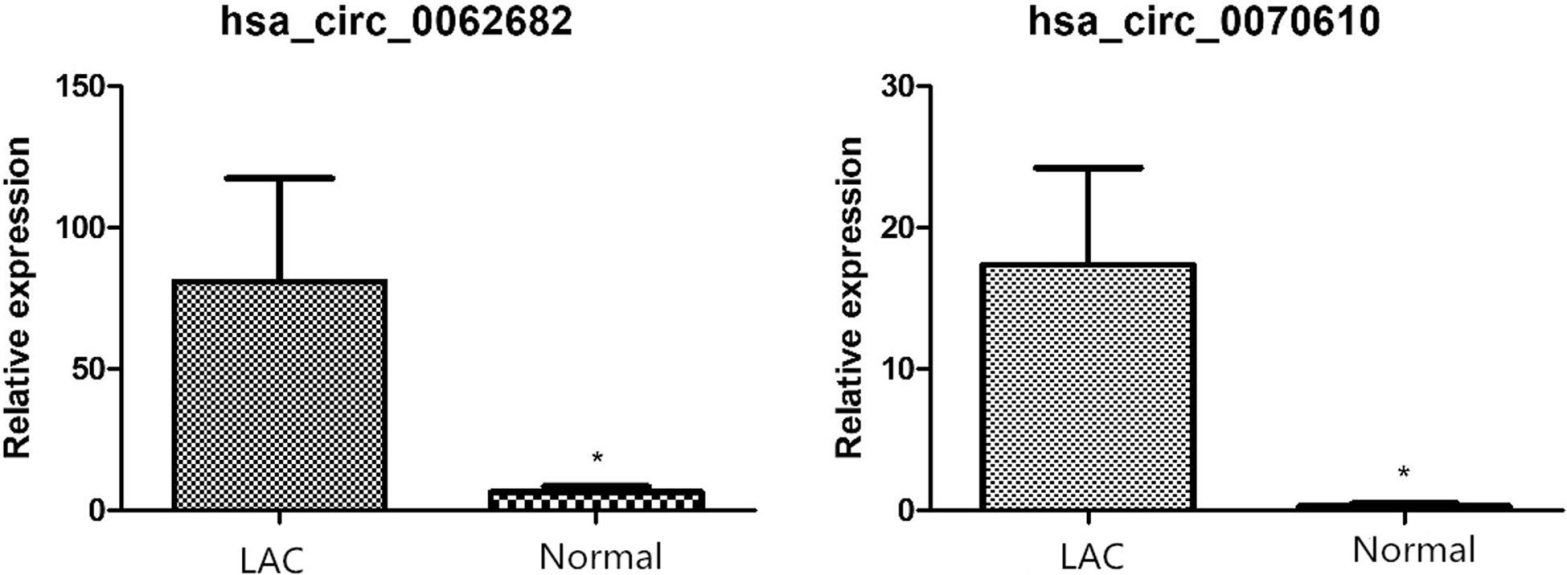
qRT-PCR validation of the expression of circRNAs

Expression of hsa_circ_0062682 and hsa_circ_0070610 was measured using qRT-PCR in 20 LAC tissues compared with paired adjacent non-tumorous tissues. As shown in Fig. 8, the expressions of hsa_circ_0062682 and hsa_circ_0070610 were significant up-regulated in LAC tissues (*P* < 0.05). However, we did not find any significant difference in the expression of LAC tissues and that of paired adjacent non-tumorous tissues for hsa_circ_0092283. In these samples, the expression abundance of hsa_circ_0092283 was very low. We may need to verify this circRNA using more samples.

## Discussion

LAC remains a main cause of cancer-related mortality owing to the high incidence and lack of effective therapy. Thus, it is important to identify potential therapeutic targets for LAC management. Existing evidence has implied that a growing number of tumour-related circRNAs have been found, and these circRNAs displayed abnormal expressions in several types of cancers based on the high-throughput sequencing analyses [21–23]. Moreover, extensive studies have shown that circRNAs functioned as ‘miRNA sponges’ and that the circRNA–miRNA–mRNA axis might participate in cancer-related pathways [24, 25]. In this study, we performed bioinformatics analysis to systematically screen the molecular markers for LAC diagnosis and treatment using a circRNA expression profile. A total of 35 DEcircRNAs (30 up-regulated DEcircRNAs and 5 down-regulated DEcircRNAs) were identified, and there were three up-regulated DEcircRNAs (hsa_circ_0062682, hsa_circ_0092283 and hsa_circ_0070610) in the circRNA–miRNA–mRNA network.

hsa_circ_0070610 was bound to two target miRNAs (hsa-let-7a-2-3p and hsa-miR-3622a-3p). Research has shown that let-7 members are involved in tumour growth and development. Yanaihara et al previously pointed out that hsa-let-7a-2 expression was dysregulated in lung cancer tissues as compared to that in non-cancer tissues; moreover, hsa-let-7a-2 underexpression was related to poor survival [26]. Moreover, *NAMPT*, *TNFSF1* and *PSMA 5* were targets of hsa-let-7a-2-3p. These genes also displayed close relationships with multiple drugs, such as teglarinad chloride, denosumab and anastrozole. Yu et al suggested that *TNFSF11* (also known as *RANKL*) expression level was elevated in patients with lung carcinoma and bone metastasis [27]. Our survival analysis showed that a lower *TNFSF11* level was markedly linked with favourable clinical outcomes in patients with LAC. NAMPT plays an essential role in NAD(+) biosynthesis in cancer cells and can regulate cellular metabolism, such as altered carbohydrate metabolism in cancer cells [28]. Further, NAMPT is an enzyme essential for NAD+ biosynthesis [28]. A number of NAMPT small molecule inhibitors have been synthesised and applied in clinical practice to date [29]. In our research, teglarinad chloride was found to be an inhibitor for NAMPT. Prostate-specific membrane antigen (PSMA) overexpression is observed in many tumours, such as prostate cancer, gliomas, lung cancer and thyroid cancer [30]. In our research, up-regulated *TNFSF11, PSM 5* and *NAMPT* were strongly associated with worse prognosis. However, the involvement of hsa-miR-3622a-3p in the molecular mechanism of LAC has not been studied.

Another circRNA hsa_circ_0092283 exhibited a close association with hsa-miR-4685-5p and hsa-miR-762. Although several trials have shown that hsa-miR-762 were responsible for the occurrence and progression of various cancers, such as bladder cancer and ovarian cancer, whether hsa-miR-4685-5p and hsa-miR-762 participated in the pathogenesis of early-stage LAC remains unknown [31, 32].

In addition, our non-coding RNA network analysis showed that hsa_circ_0062682 closely interacted with hsa-miR-4268. A previous study examined the expression change of miRNAs in patients with metastatic melanoma before and after surgical resection; the results indicated that hsa-miR-4268 had a differential expression [33]. Zhao et al recently showed that hsa-miR-4268 overexpression inhibited the cell proliferation and induced cell apoptosis of gastric cancer cells, suggesting that hsa-miR-4268 might be a tumour suppressor in the development of gastric cancer [34]. However, the direct influences of miR-4268 on LAC progression were being researched at the time of writing this report.

The functional analyses of genes in the circRNA–miRNA–mRNA network revealed that they were predominately correlated with the Wnt signalling pathway and positive regulation of GTPase activity. Activation of the Wnt signalling pathway can promote lung cancer progression and contribute to poor patient prognosis [35]. Many studies have proved that constitutive activation of Wnt/β-catenin signalling plays an important role in LAC development [36].

However, this research has certain limitations. First, an integrated bioinformatics analysis needs to be performed on the basis of multiple circRNA microarray datasets to confirm our results. Second, the additional experimental assays were required to validate the results from bioinformatics analyses. Third, the comprehensive clinical information should also be integrated into a large-scale survival analysis. Finally, the potential interactions of non-coding RNAs (circRNA/miRNA/mRNA) need further elaboration in the future.

In sum, three circRNAs (hsa_circ_0062682, hsa_circ_0092283 and hsa_circ_0070610) and five miRNA targets (hsa-miR-762, hsa-miR-4685-5p, hsa-let-7a-2-3p, hsa-miR-4268 and hsa-miR-3622a-3p) were probably associated with the development of early-stage LAC. Moreover, *PSMA 5*, *TNFSF11*, *NAMPT* and *CPT2* acted as promising diagnostic and prognostic makers for LAC management.

## Data Availability

The expression data were downloaded from the National Centre for Biotechnology Information Gene Expression Omnibus (NCBI-GEO) repository (http://www.ncbi.nlm.nih.gov/geo/). In our research, we used the dataset GSE101684.

## Abbreviations

NSCLC: Non-small cell lung cancer
circRNAs: Circular RNAs
ceRNAs: Competitive endogenous RNAs
DEcirRNAs: Differentially expressed circRNAs
NCBI-GEO: National Center for Biotechnology Information Gene Expression Omnibus
TCGA: The Cancer Genome Atlas
